# Evaluate the integrative effects of irrigation water level, furrow irrigation methods, and nitrogen fertilizer rate on tomato yield in semi-arid southern Ethiopia

**DOI:** 10.1016/j.heliyon.2024.e41551

**Published:** 2024-12-27

**Authors:** Edmealem Temesgen Ebstu, Mekuanent Muluneh

**Affiliations:** a Faculty of Water Resources and Irrigation Engineering, Arba Minch Water Technology Institute, Arba Minch University, Arba Minch, Ethiopia; b Water Resources Research Center, Arba Minch Water Technology Institute, Arba Minch University, Arba Minch, Ethiopia

**Keywords:** Integrated effects, Irrigation water, Nitrogen fertilizer, Tomato yield, Furrow irrigation, Southern Ethiopia

## Abstract

This study investigates the integrative effects of irrigation water management allowable depletion (MAD), furrow irrigation methods (FIM), and nitrogen fertilizer application rate (NFAR) on tomato yield components. These yield components include marketable, unmarketable, and total yield. Additionally, the study examines crop agronomy components such as plant height, number of branches, and root depth in semi-arid Southern Ethiopia. The research employs a factorial and split-plot design. Three types of FIM are assigned as the main plot, while three levels of irrigation water (50 %, 75 %, and 100 % MAD) and four levels of NFAR (0 %, 50 %, 100 %, and 150 % NFAR) serve as the subplots, with three replications. The study results reveal significant impacts of these factors. All p-values for tomato yield and agronomy-measured components are less than 0.05. FIM, irrigation water level, and NFAR interaction affect marketable, unmarketable, and total tomato yield. The results ranged from 2.3 tons ha⁻^1^–43.9 tons ha⁻^1^ for marketable yield, 0.8 tons ha⁻^1^–8 tons ha⁻^1^ for unmarketable yield, and 6.3 tons ha⁻^1^–45.2 tons ha⁻^1^ for total yield. The integration of 75 % MAD with 100 % NFAR under conventional furrow irrigation, and 100 % MAD with 100 % NFAR under alternative furrow irrigation, was most preferable. This approach resulted in no loss of nitrogen fertilizer and saved 25 % of irrigation water without reducing tomato yield. However, the interaction of deficit nitrogen fertilizer rate application, with or without full application of MAD and conventional or other furrow irrigation methods, caused the total yield to decrease. CFIM and AFIM with 100 % MAD and 100NFAR treatments yielded the highest profits, while the FFIM approach under 50%MAD and 0%NFAR conditions negatively impacted profitability. Therefore, by adopting the preferable practices, farmers can achieve higher productivity and sustainability in tomato cultivation. This approach addresses the challenges posed by water scarcity and nutrient limitations.

## Highlights

• Optimal irrigation methods, water levels, and nitrogen rates for Maximum Yield.
• Optimizing irrigation and nitrogen to improve tomato yield in semi-arid regions.
• Integrated management practices improved overall plant health and resilience.
• Integrating irrigation and nitrogen for sustainable tomato yield in water-scarce.
• Adequate irrigation and nitrogen application minimized unmarketable fruits.

## Graphical abstract

**Figure:**
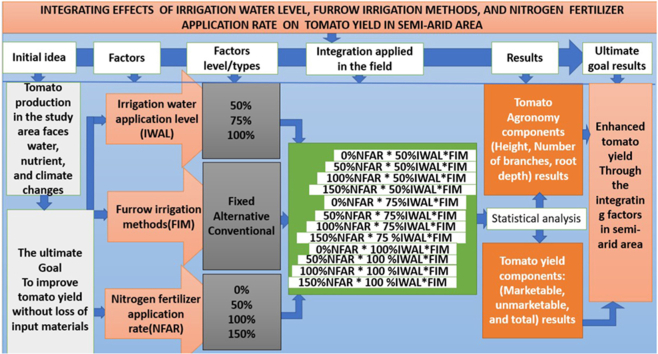

## 1 Introduction

Tomato (Solanum lycopersicum L.) production plays a vital role in the Ethiopian economy, particularly for smallholder farmers in semi-arid Arba Minch regions in southern Ethiopia [1]. The crop is highly valued for its nutritional content as a vitamin source and is a vital source of income for farmers [2]. However, the sustainability of tomato cultivation faces significant challenges due to the region's semi-arid climate. Sustainable agriculture in this region requires innovative strategies to optimize resource use and enhance productivity. Limited water resources and inefficient irrigation practices threaten crop yields.

Water scarcity is a prevalent issue in many arid and semi-arid regions of Ethiopia, particularly Arab Minch Southern Ethiopia [3]. Irrigation agriculture, which accounts for a major of water use, is heavily impacted by water shortages. Efficient water management practices are crucial for ensuring irrigation agricultural sustainability and food security [4]. The need for efficient water management strategies is paramount. Farmers can maintain acceptable tomato crop yields by applying optional water-reduced levels, and furrow irrigation methods. Irrigation water level deficit has emerged as a viable strategy, to cope with water scarcity [5].

Irrigation water level deficit involves applying water below the crop's full evapotranspiration requirement, which can lead to significant water savings without substantially reducing yields [6]. This method conserves water and encourages deeper root growth, enhancing the plant's ability to access soil moisture. Irrigation water level deficit has emerged as a promising approach. Irrigation water level deficit involves applying a controlled water deficit below the crop's full evapotranspiration (ETc) requirements during specific growth stages [7]. This strategy can stimulate root development, enhance plant tolerance to drought stress, and improve water use efficiency [8-9]. Studies have shown that strategically implemented Irrigation water level deficit can maintain or even increase tomato yields while significantly reducing water use [7]. Previous studies have shown that deficit irrigation can enhance water use efficiency and improve crop resilience to drought conditions [[10], [11], [12]].

Effective irrigation management is essential for mitigating water scarcity and maximizing crop productivity in semi-arid environments [13]. Furrow irrigation, a common method in the region, involves channeling water along furrows between crop rows, aiming to deliver water directly to the plant roots while minimizing losses to evaporation and runoff [13]. However, the efficiency of this method can vary significantly based on how it is implemented. Modified methods, such as alternate furrow or fixed irrigation, aim to improve water distribution and reduce evaporation losses [14]. However, Arba Minch's semi-arid region uses traditional conventional furrow irrigation methods, often resulting in reduced water wastage, low Water use efficiency, and reduced crop yield. Smart irrigation can improve irrigation decisions, save water, and increase yields in semi-arid and arid areas. Methods such as artificial intelligence, deep learning, model predictive irrigation systems, variable rate irrigation (VRI) technology, and unmanned aerial vehicles (UAVs) enhance water use efficiency in water-scarce regions [[15], [16], [17]]. These technologies improve water management practices and advance progress toward multiple Sustainable Development Goals (SDGs).

Nitrogen (N) management is another crucial factor for sustainable tomato production [18]. It is a vital nutrient for plant growth and development, tomato yield, and fruit quality, by playing a key role in photosynthesis and protein synthesis. However, its management is challenging due to its excessive or deficit nitrogen application, high mobility in the soil, and susceptibility to losses through leaching and volatilization [19]. Additionally, excessive nitrogen application not only increases production costs but also poses significant environmental risks. These risks include groundwater contamination, eutrophication, ecosystem degradation, algae blooms in water bodies, harm to human health, and increased greenhouse gas emissions [20]. Moreover, not only excessive but also, a deficit of nitrogen fertilizer dose is the main factor that reduces the yield and quality of crops. Applying the recommended nitrogen rate based on soil analysis can ensure adequate plant nutrition. Utilizing slow-release fertilizers or implementing split applications throughout the growing season can also help. These methods minimize environmental impact while promoting healthy plant growth [[21], [22], [23]]. Therefore, precise nitrogen management is essential to maximize crop productivity and indirectly minimize environmental impact.

The interaction between irrigation water levels, furrow irrigation methods, and nitrogen fertilizer rates is complex and critical for optimizing crop yield performance. Previous research has extensively studied the individual impacts of irrigation water levels, furrow irrigation methods, and nitrogen fertilizer rates on crop yield, but there is limited understanding of their interactive effects [[21], [22], [23]]. Research on deficit irrigation and nitrogen management for tomato production has been conducted globally, yielding promising results. Studies have demonstrated the effectiveness of irrigation water level deficit in improving water use efficiency (WUE) while maintaining or increasing yields in various regions [[10], [11], [12]]. Likewise, experiments investigating the optimal application strategies for nitrogen have shown positive results on the yield, the fruit quality, and the sustainability of the environment [20]). The interaction between these factors can significantly influence crop performance, water use efficiency, and nutrient uptake [19]. However, it is essential to evaluate the impacts of irrigation water level deficits, different irrigation technologies, and nitrogen fertilizer interaction practices. This evaluation is particularly important in distinct environments and agricultural circumstances in semi-arid regions.

Arba Minch, located in southern Ethiopia, is well-known for its semi-arid climate characterized by limited and unpredictable rainfall [3]. Agriculture in this region is predominantly rain-fed, making it vulnerable to droughts and climate variability [24]. The region's socio-economic context, where agriculture is a primary source of livelihood, highlights the urgency of improving agricultural practices to ensure food security and enhance farmer incomes [25]. Therefore, understanding these interactions is crucial for developing integrated management practices for water and nitrogen applications. This approach aims to overcome challenges related to sustainability and optimization of tomato crop production in the region. This study addresses this knowledge gap by investigating the interactive effects of varying irrigation water levels, furrow irrigation methods, and nitrogen fertilizer rates on tomato yield, quality, resource use efficiency, and overall sustainability of tomato production in Southern Ethiopia.

## 2 Materials and methods

### 2.1 Description of the study area

**Fig. 1:**
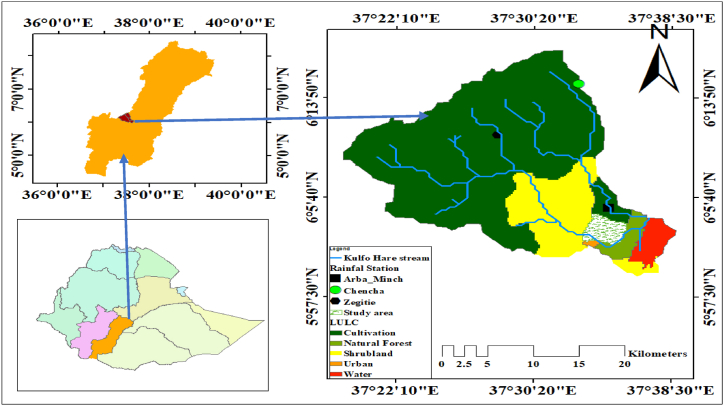
The study area location Map.

The study area was conducted between Kulfo and Hare Rivers, on the eastern side of Arba Minch University, and extends towards Lake Abaya at the demonstration farm of Arba Minch University. The geographic location of the site was 6°05′ N Latitude, 37°34′ E Longitude (Fig. 1), and an elevation of 1203-m above mean sea level. The climate of Arba Minch is typically semi-arid, with a hot and extremely arid winter season. During the summer and alternative rainy seasons, the weather conditions are characterized by mild or fair weather. Long-term weather data from the Arba Minch meteorological station shows that the study area's maximum temperature is 33.33C and the minimum temperature is close to 17.4 °C. The average relative humidity varies from 73 % (May) to 58 % (January), the annual daily sunshine duration is 8.9 to 7.9 h and is characterized by low annual rainfall (400–600mm) and high evapotranspiration rates. Samples of soil were taken randomly from the experimental field. The samples were taken from depths 0–90cm within 30m depth intervals. The average measured values of clay, silt, and sand in percentages were 28.61, 25.88, and 38.76 %, respectively and the soil texture is Clay loam. The average bulk density of the site was 1.31g/cm^3^.

### 2.2 Experimental treatment and design

The study experimental treatment consists of three furrow irrigation technique types (conventional, alternative, and fixed furrow irrigation) with three irrigation water management allowable depletion (MAD) (50 %, 75 %, and 100 % MAD) and four N fertilizer application rates(NFAR) (0, 50, 100 %, and 150 %NFAR. These treatments were applied over two consecutive years, in 2021 and 2022 (Table 1). Management allowable depletion (MAD) refers to the maximum amount of soil water the irrigation manager permits the crop to extract from the active rooting zone before the next irrigation. Three furrow irrigation techniques are conventional, fixed, and alternate. There was a total of 36 treatment combinations (four N fertilizer application rates, and three irrigation water levels) with 3 replications for each three furrow irrigation types.

**Table 1:** Experimental study treatment variables.

Treatments	CFI	Treatments	AFI	Treatments	FIM
	Factors of Treatment		Factors of Treatment		Factors of Treatment
T1	100 % MAD with 0%NFAR	T13	100 % MAD with 0%NFAR	T25	100 % MAD with 0%NFAR
T2	75 % MAD with 0%NFAR	T14	75 % MAD with 0%NFAR	T26	75 % MAD with 0%NFAR
T3	50 % MAD with 0%NFAR	T15	50 % MAD with 0%NFAR	T27	50 % MAD with 0%NFAR
T4	100 % MAD with 50%NFAR	T16	100 % MAD with 50%NFAR	T28	100 % MAD with 50%NFAR
T5	75 % MAD with 50%NFAR	T17	75 % MAD with 50%NFAR	T29	75 % MAD with 50%NFAR
T6	50 % MAD with 50%NFAR	T18	50 % MAD with 50%NFAR	T30	50 % MAD with 50%NFAR
T7	100 % MAD with 100%NFAR	T19	100 % MAD with 100%NFAR	T31	100 % MAD with 100%NFAR
T8	75 % MAD with 100%NFAR	T20	75 % MAD with 100%NFAR	T32	75 % MAD with 100%NFAR
T9	50 % MAD with 100%NFAR	T21	50 % MAD with 100%NFAR	T33	50 % MAD with 100%NFAR
T10	100 % MAD with 150%NFAR	T22	100 % MAD with 150%NFAR	T34	100 % MAD with 150%NFAR
T11	75 % MAD with 150%NFAR	T23	75 % MAD with 150%NFAR	T35	75 % MAD with 150%NFAR
T12	50 % MAD with 150%NFAR	T24	50 % MAD with 150%NFAR	T36	50 % MAD with 150%NFAR

Note: MAD is management allowable depletion and NFAR is nitrogen fertilizer application rate.

**Fig. 2:**
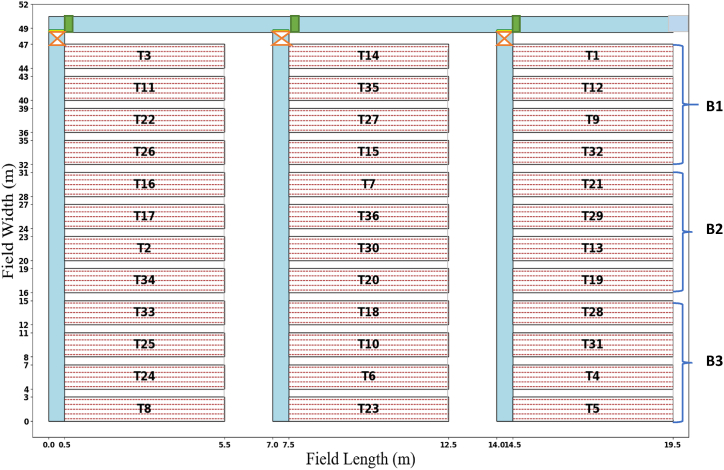
Experimental field plot layout. Note: B1, B2, and B3 are block1, block2, block3 respectively. The blue line is irrigation water in the canal; all dimensions are in meters.

The experimental field was plowed and harrowed to achieve a fine tilth, which is essential for good root development and irrigation efficiency. The experiment was arranged in a split-plot with a factorial subplot. The furrow irrigation methods factor is assigned to the main plots, and combinations of factors N fertilizer level and irrigation water levels are assigned to a subplot designed with three replicates for each treatment combination. The plot size was 3m × 5m, and the distance between replications and plots was 1.5m and 1m, respectively (Fig. 2). Moreover, the spacing between rows and plants was 30 cm and 30 cm, respectively. The land was plowed two times and prepared for planting using manpower. The total area of the experimental field was 916.5 m^2^ (47m × 19.5m) and the Field Experimental layout for this research is shown in Fig. 2.

### 2.3 Tomato seed rate and nursery establishment

Eight-gram Roma variety packed tomato (Solanum lycopersicum, L) seeds suitable for semi-arid environments were sown in a prepared seedbed (2m × 4m) (8m^2^) on October 1, 2020, and for the second round on October 1, 2021. The bed was well irrigated before sowing. Subsequently, the bed was irrigated regularly and shaded properly until the seedlings were ready for transplanting.

The shade was provided to protect the seedlings from high temperatures and heavy downpours using grass materials. The seedling management practice was followed until the seedlings reached the transplant stage. The first-round seedlings were then transplanted on November 1, 2020, and for the second round on November 1, 2021, on well-prepared experimental plots, which means it was done when the tomato reached 10 cm in height and had a good, sturdy stem.

### 2.4 Crop water requirement

The crop water requirement (CWR) to balance the quantity of water lost through evapotranspiration (ETc), is calculated from reference evapotranspiration (ETo) and tomato crop coefficient (Kc) [26]. The water requirement of the crop (ETc) was then calculated using CROPWAT software over the growing period from ETo and the crop coefficients (Kc)using Equation (1).

(1) ETc=ETo∗Kc

Where: ETo is reference crop evapotranspiration (mm/day); ETc is crop evapotranspiration (mm/day), and Kc is Crop coefficient.

The reference evapotranspiration (ETo) was calculated using the modified FAO Penman-Monteith calculation based on the daily record of climatic data [26] using FAO CROPWAT software version 8.0.and using Equation (2). The input data for the CROPWAT software includes. Maximum and minimum temperatures, relative humidity, sunshine duration, and wind speed.

(2) $$\text{ETo}=\frac{0.408\left(\text{Rn}-G\right)+\Upsilon \frac{900}{T+273}U2\left(\text{es}-\text{ea}\right)}{\Delta +\Upsilon \left(1+0.34U2\right)}$$

Where; ETo is Reference crop evapotranspiration (mm); Δ is Slope of vapor pressure curve (kPa/°C); Rn is Net radiation at the crop surface (MJ/m/d); G is Soil heat flux density (MJ/m/d); T is Air temperature at 2m height (°C); Es is Saturation vapor pressure (kPa); Ea is Actual vapor pressure (kPa); es - ea is Saturation vapor pressure deficit (kPa); u2 is Wind velocity at 2 m height (m/s); γ is Psychometric constant (kPa/°C).

We used the Kc values of tomatoes planted 0.89, 1.22, 0.97, and 0.64 in the late, mid, development, and initial seasonal stages respectively [27].

The net requirement of irrigation (IRn) (mm) was calculated using the CROPWAT software based on [26] as shown in Equation (3).

(3) IRn=ETc−Pe

Where: ETc crop water requirement (mm), and Pe is effective rainfall (mm) which is part of the rainfall that inserts into the soil and makes it available for crop production.

Effective rainfall (Pe) was estimated using the empirical formula. An empirical formula developed by FAO based on analysis for different arid and sub-humid climates as given in Equation (4) or Equation (5) [28].

(4) Pe=0.6∗TotalRainfall‐10,(TotalRainfall<70mm)

(5) Pe=0.8∗TotalRainfall‐24,(TotalRainfall>70mm)

The gross irrigation requirements account for losses of water incurred during conveyance and application in the irrigation site. The total irrigation requirement was computed by adopting a field application efficiency of 60 % because the experiment was done at the research center site. This is stated in terms of efficiencies when calculating project gross irrigation requirements from net irrigation requirements, as shown below in Equation (6) [28].

(6) $$\text{IRg}=\frac{\text{IRn}}{\text{Ea}}$$

Where: IRg is gross irrigation requirement (mm); IRn is net irrigation and Ea is irrigation efficiency.

The interval of irrigation is the time gap between two consecutive irrigation applications in days. It depends on the crop type, soil type, and climate conditions. Thus, the interval of irrigation depends on the crop consumptive level and the quantity of readily available moisture (RAM) in the root of the crop. The CROPWAT software computes the CWR, ET_0_, and IRs to compute the schedules of irrigation under various administration situations and water application plans using Equation (7) [28].

(7) $$i\left(\text{days}\right)=\frac{A_{s}.D\left(\text{FC}\;‐\;\text{PWP}\right)\;.\;P}{E{T_{\text{crop}}}_{\left(\text{peak}\right)}}$$

Where: i is irrigation interval; As is soil apparent specific gravity; D is Effective root depth in m; FC is water content of soil at FC; PWP is Water content of soil at PWP; P is depletion factor; ETc is crop evapotranspiration (mm day^−1^).

The irrigation was started one week after transplanting and ended for all treatments six days before the last harvest. The time required to deliver the required quantity of water into each furrow of the experimental plot was planned using Equation (8) [26].

(8) $$T=\frac{\text{GIR}*L*w}{60*Q}$$

Where: T is Application time (min); GIR is Gross level of water applied (mm); l is Furrow length in (m); w is Furrow spacing in (m); and Q is Flow level (l s^−1^).

The water conveyed to the experimental field through an unlined open channel was measured. By using a calibrated circular pipe. The pipe was calibrated to a discharge of 2.1Ls^−1^ and the constant head on the sheet metal gate was marked to control the variation. The corresponding quantity of water was used for different treatments using a blocked different furrow irrigation method. The field irrigation canals up to the furrow inlet were roofed with a plastic membrane to control infiltration losses in the field channel.

### 2.5 Furrow irrigation methods

The experiment employed three distinct furrow irrigation methods.

1 Conventional Furrow Irrigation: In this method, every furrow in the plot was irrigated during each irrigation event. This method ensured uniform water application to the entire crop throughout the growing season, providing continuous moisture to the plants.
2 Alternate Furrow Irrigation: In this method, irrigation was applied alternately to every other furrow during each irrigation cycle. This approach aimed to improve water-use efficiency by allowing non-irrigated furrows to dry between irrigation cycles while maintaining moisture in the root zone of the irrigated furrows.
3 Fixed Furrow Irrigation: The fixed furrow method followed the same principle as alternate furrow irrigation but maintained the same set of furrows irrigated throughout the entire growing season. This method also sought to enhance water efficiency by concentrating irrigation efforts on specific furrows, reducing water loss through evaporation and deep percolation while maintaining a consistent irrigation pattern.

Each furrow irrigation method was combined with three different management allowable depletion (MAD) levels 50 %, 75 %, and 100 %, and four nitrogen fertilizer application rates 0 %, 50 %, 100 %, and 150 % of the recommended nitrogen fertilizer application rate (NFAR). These combinations created a total of 12 treatment variations for each furrow irrigation method, as outlined in Table 1.

### 2.6 Nitrogen fertilizer application strategy

Nitrogen fertilizer (urea) was applied at specified four different rates based on the treatment requirements of 0 % NFAR (control), 50 % NFAR, 100 % NFAR (recommended rate of 210 kg/ha) [29], and 150 % NFAR**.** The time of nitrogen application rate was split into two stages during the tomato growth season to ensure efficient uptake and to avoid nitrogen losses due to leaching or volatilization [29].

The first half of the required nitrogen dose was applied at the time of transplanting**.** This application took place within one week after transplanting the seedlings to support the early vegetative growth of the plants. Adequate nitrogen at this stage is crucial for strong root and shoot development. Second Nitrogen Application time**:** The remaining half of the nitrogen dose was applied during the early flowering stage**,** approximately 35–40 days after transplanting**.** This application aimed to supply the nitrogen necessary for fruit set and development, ensuring sufficient nitrogen availability during the reproductive phase of the plant's growth.

Nitrogen was applied using a side-dressing technique, placing the fertilizer along the rows of plants instead of directly at the base of each plant. This method reduced the risk of nitrogen loss and ensured that the nutrient was available within the active root zone. In addition to nitrogen, basal doses of phosphorus (160 kg/ha) and potassium (320 kg/ha) were applied uniformly across all plots to ensure that these essential nutrients were not limiting and that the focus of the study remained on the effect of nitrogen and irrigation treatments [30].

### 2.7 Agronomic practices

Standard agronomic practices, including weeding, pest control, and disease management, were uniformly applied to all plots. Weeding was carried out manually at regular intervals to control weed competition, which can affect tomato growth and yield. Regular monitoring was done for common tomato diseases such as blight, wilt, and leaf spot. Appropriate measures were taken to control outbreaks promptly. Tomato plants were staked using wooden stakes and twine to support the plants, keep fruits off the ground, and reduce the risk of disease. Regular pruning of side shoots and lower leaves was done to improve air circulation, reduce disease incidence, and promote better fruit development.

### 2.8 Data collection

Crop components refer to the individual factors that contribute to the total production of a crop. These factors typically include the plant height, the number of branches per plant, and the root depth. Understanding yield components helps identify which aspects can be optimized to increase crop yield. Tomato crop parameters such as the number of branches, plant height, and root depth were measured during different stages of growth. Some tomato plants were randomly taken from the middle rows of the experimental plots to collect data on plant height, branch number, and root depth. The plant height (cm) and branch number were measured regularly throughout the growing season and their average values were considered for further statistical analysis. The plant height was measured from the surface of the soil to the tip of the leaves of randomly selected plants from the center of two rows of each plot after 75 days of transplanting. Root depth(cm) was measured by using a digital caliper at each harvesting time, and their average value was considered for further analysis using a root cuter instrument.

The crop yield components namely marketable fruit, non-marketable fruit, and total tomato yield were collected from each treatment's center ridge (row). Tomato fruit yields were harvested at maturity from each plot. Fruits were carefully hand-picked to avoid damage. Harvesting was done in the early morning to minimize heat stress on the fruits. The yield was measured in terms of total fruit weight per plot. The total weight of tomatoes harvested from each plot was recorded to determine the yield.

### 2.9 Economic analysis approach

We followed a structured approach that calculated total costs, revenues, and profitability for each treatment to perform the economic analysis of the different tomato production treatments. The economic feasibility of each treatment was assessed using the following equations (9), (10), (11)(31].

#### 2.9.1 Total Input Cost calculation

(9) TotalInputCost($)=FertilizerCost+LaborCost+SeedCost

Where: Fertilizer Cost is the Cost associated with nitrogen fertilizer application, Labor Cost is the Cost including land preparation, digging, weeding, and harvesting, Seed Cost is the Cost of seeds used for planting.

#### 2.9.2 Total Revenue Calculation

(10) TotalRevenue=Yield×PriceperUnit

Where: Yield is the total output produced per treatment, and price per Unit is the selling price of the tomato yield.

#### 2.9.3 Profit calculation

(11) Profit=TotalRevenue‐TotalInputCost

This equation indicates the net profit derived from each treatment after accounting for all associated costs.

### 2.10 Statistical analysis

The experimental data were analyzed using a three-way analysis of variance (ANOVA) to examine the effects of irrigation water levels, furrow irrigation methods, and nitrogen fertilizer rates on tomato yield and associated agronomic parameters. The study employed a factorial split-plot design, where irrigation water levels were assigned to the main plots, furrow irrigation methods, and nitrogen fertilizer rates were allocated to the sub-plots. This design allowed the assessment of interaction effects between the three factors. The three-way ANOVA model used for the analysis [32]is expressed in Equation (12).

(12) $$Y_{\text{ijkl}}=\mu +I_{i}+F_{j}+N_{k}+{\left(I\times F\right)}_{\text{ij}}+{\left(I\times N\right)}_{\text{ik}}+{\left(F\times N\right)}_{\text{jk}}+{\left(I\times F\times N\right)}_{\text{ijk}}+\epsilon_{\text{ijkl}}$$

Where: Y_ijkl_ is tomato yield, μ is the overall mean, I_i_ is the effect of the i_th_ irrigation level, F_j_ is the effect of the j_th_ furrow irrigation method, N_k_ is the effect of the k_th_ nitrogen fertilizer rate, and ϵ_ijkl_ is the random error.

The analysis of variance (ANOVA) was used to evaluate the main and interaction effects. All significant effects (p < 0.05) were further explored using post-hoc tests. The least significant difference (LSD) test was used to compare treatment means, while Tukey's Honest Significant Difference (HSD) test was applied for pairwise comparisons where significant differences were found. These post-hoc tests helped identify which specific treatment combinations significantly affected tomato yield and crop parameters.

To ensure the validity of the results, the assumptions of ANOVA were tested. The normality of residuals was checked using the Shapiro-Wilk test, and the homogeneity of variances was assessed using Levene's test. When necessary, data transformations were applied to meet these assumptions. The statistical analyses were conducted using SAS statistical software (Version 9.4), which ensured precision in handling the split-plot design and reproducibility of the study.

## 3 Results

### 3.1 Tomato crop agronomy components

The factors significantly influenced the plant height, number of branches, and root depth of tomato crops. The effects of the integrating factors were statistically significant, with a p-value of less than 0.05, as shown in Table 2.

**Table 2:** Interaction effects of considered factors on plant height, number of branches, and root depth of tomato crop.

Treatments	Conventional furrow irrigation method (CFIM)			Alternate furrow irrigation method (AFIM)			Fixed furrow irrigation method (FFIM)		
	Plant Height (cm)	No. Branch	Root depth(cm)	Plant Height (cm)	No. Branch	Root depth (cm)	Plant Height (cm)	No. Branch	Root depth(cm)
100%MAD ∗ 0%NFAR	65^d^	4^def^	20.9^p^	48.8^j^	3^hij^	15.74^z^	43.9^n^	3^ijk^	14.2^B^
100 % MAD ∗ 50%NFAR	72^c^	5^cd^	30.9^f^	54.0^h^	3^hij^	23.24^n^	48.6^k^	3^ijk^	20.9^q^
100%MAD ∗ 100%NFAR	83^a^	8^a^	45.9^a^	62.3^e^	6^b^	34.5^d^	56.0^g^	6^bc^	31.0^e^
100%MAD ∗ 150%NFAR	75^b^	5^bc^	39.9^b^	56.3^f^	4^defg^	29.99^g^	50.6^i^	4^efghi^	26.9^i^
75%MAD ∗ 0%NFAR	48.75^j^	4^def^	15.9^y^	36.6^u^	3^hij^	11.99^F^	32.9^x^	3^ijk^	10.9^G^
75%MAD ∗ 50%NFAR	54^h^	4^def^	23^l^	40.5^r^	3^hij^	17.99^u^	36.5^v^	3^ijk^	16.2^x^
75%MAD ∗ 100%NFAR	62.3^e^	7^b^	34.9^c^	46.7^m^	5^de^	26.24^j^	42.0^q^	4^efghi^	23.6^m^
75%MAD ∗ 150%NFAR	56.25^f^	5^cd^	28.9^h^	42.2^p^	4^defg^	21.74^o^	37.9^s^	3^ijk^	19.6^s^
50%MAD ∗ 0%NFAR	36.6^t^	3^ghij^	9.9^H^	27.4^c^	2^ghij^	7.49^l^	24.7^e^	2^k^	6.7^J^
50%MAD ∗ 50%NFAR	40.5^r^	4^def^	17.9^v^	30.4^A^	3^hij^	13.49^D^	27.34^D^	3^ijk^	12.1^E^
50%MAD ∗ 100%NFAR	46.7^l^	4^def^	25.9^k^	35.0^w^	3^hij^	19.49^t^	31.5^z^	3^ijk^	17.5^w^
50%MAD ∗ 150%NFAR	42.2^o^	4^def^	19.9^r^	31.6^y^	3^hij^	14.99^A^	28.5^b^	3^ijk^	13.5^C^
CV (%)	0.05	15.5	0.08	0.05	15.5	0.08	0.05	15.5	0.08
Sem ±	0.013	0.35	0.01	0.013	0.35	0.01	0.013	0.35	0.01
Grand mean	45.96	3.96	21.3	45.96	3.96	21.28	45.96	3.96	21.3
FIM∗(IWAL∗NFRA) (P)	0.0001	0.01	0.01	0.0001	0.01	0.0001	0.0001	0.01	0.01

**Note:** Mean results in the same column followed by different letters are significantly different in the least significant difference (LSD) tests at *P* < 0.05 level, NFAR is Nitrogen fertilizer application rate, and MAD is management allowable depletion IWAL.

The plant height ranged from 24.7 cm to 83 cm due to the interaction of all factors in the different treatments. The tallest tomato crop, 83 cm, was observed in the control treatment (T7), which involved 100 % MAD with 100 % NFAR under conventional furrow irrigation methods (CFIM). Following the control, the tallest plant heights were recorded in treatments 100 % MAD with 150 % NFAR under CFIM (75 cm), 100 % MAD with 50 % NFAR under CFIM(72 cm), 100 % MAD with 0 % NFAR under CFIM(65 cm). While, the shortest plant height, 24.7 cm, was observed in treatment T27, which consisted of 50 % MAD with 0 % NFAR under fixed furrow irrigation methods (FFIM) (Table 2).

The mean number of tomato branches varied from 2 to 8 depending on the furrow irrigation method (FIM), management allowable depletion (MAD), and N-fertilizer application rate (NFAR). The highest mean number of branches (8) was observed in the conventional furrow irrigation method (CFIM), with 100 % MAD and 100 % NFAR (T7). Following T7, the three treatments with the maximum number of branches were 75 % MAD, 100 % NFAR, and CFIM (T8); 100 % MAD, 100 % NFAR, and alternative furrow irrigation (T19); and 100 % MAD, 100 % NFAR, and fixed furrow irrigation (T31). On the other hand, the minimum number of branches (3) was recorded in the treatment of 50 % MAD and 0 % NFAR under alternative and fixed furrow irrigation methods (T27) (Table 2).

The study findings revealed that the root depth of tomato crops varied between 6.7 cm and 45.9 cm. The control group, which utilized conventional furrow irrigation systems with 100 % MAD and 100 % N nitrogen fertilization rate, exhibited the highest root depth of 45.9 cm. Following the control treatment, the three treatments with the highest root depths per plant were 100 % MAD with 150 % NFAR under CFI, 100 % MAD with 100 % NFAR under CFI, and 75 % MAD with 100 % NFAR under AFI. These treatments' root depth displayed values were 39.9 cm, 34.9 cm, and 34.5 cm respectively. The lowest recorded root depth per plant was 6.7 cm, which was observed in the treatments using fixed furrow irrigation systems with 50 % MAD and 0 % nitrogen fertilization application rate (Table 2).

### 3.2 Integrated effects on tomato yield components

The integration of these factors significantly influenced the yield components of tomatoes, including marketable, unmarketable, and total yield, with a p-value of less than 0.05, as presented in Table 3. This indicates that the interaction of irrigation water levels, furrow irrigation methods, and nitrogen fertilizer application rates had a statistically significant effect on tomato yield.

**Table 3:** Interaction effects of the considered factors on unmarketable (UMY), marketable (MY), and total yield (TY) (tons ha^−1^).

Treatments	Conventional furrow irrigation method (CFIM)			Alternate furrow irrigation method (AFIM)			Fixed furrow irrigation method (FFIM)		
	UMY	MY	TY	UMY	MY	TY	UMY	MY	TY
100%MAD ∗ 0%NFAR	4.4^bced^	20.6^hi^	25^i^	3.3^def^	15.5^lm^	18.8^o^	3^cdefg^	13.9^mn^	16.9^r^
100 % MAD ∗ 50%NFAR	3.1^cdef^	26.9^d^	30^e^	2.4^fgh^	20.1^hij^	22.5^l^	2.1^efghi^	18.1^jk^	20.3^m^
100%MAD ∗ 100%NFAR	1.2^jk^	43.9^a^	45.2^a^	0.9^k^	32.9^b^	33.9^c^	0.8^k^	29.7^c^	30.5^d^
100%MAD ∗ 150%NFAR	1.3^jk^	33.5^b^	34.8^b^	1^k^	25.1^de^	26.1^g^	0.9^k^	22.6^fgh^	23.5^j^
75%MAD ∗ 0%NFAR	5.5^abcd^	13.2^mn^	18.8^o^	4.2^bcd^	9.9^op^	14.1^t^	3.7^bcdef^	8.9^pq^	12.7^w^
75%MAD ∗ 50%NFAR	4.8^bcde^	17.7^jkl^	22.5^l^	3.6^cde^	13.3^mn^	16.9^r^	3.2^bcdef^	11.9^no^	15.2^s^
75%MAD ∗ 100%NFAR	2.3^fghij^	31.6^bc^	33.9^c^	1.7^ijk^	23.7^efg^	25.4^h^	1.5^hijk^	21.3^gh^	22.9^k^
75%MAD ∗ 150%NFAR	1.7^ghij^	24.4^def^	26.1^f^	1.3^jk^	18.3^ijk^	19.6^n^	1.2^jk^	16.4^kl^	17.6^p^
50%MAD ∗ 0%NFAR	8.0^a^	4.3^tu^	9.4^A^	6.0^abc^	3.2^u^	7.0^E^	5.4^abcd^	2.3^u^	6.3^F^
50%MAD ∗ 50%NFAR	6.6^ab^	6.1^rst^	11.3^y^	4.9^abc^	4.6^stu^	8.4^C^	4.5^abcd^	4.1^tu^	7.6^D^
50%MAD ∗ 100%NFAR	4.6^bcde^	12.3^no^	16.9^o^	3.5^def^	9.3^pq^	12.7^v^	3.1^cdef^	8.3^pqr^	11.4^x^
50%MAD ∗ 150%NFAR	6^abc^	6.9^qrs^	13.1^u^	4.6^bcd^	5.1^stu^	9.8^z^	4.2^bcde^	4.63^stu^	8.8^B^
CV (%)	56	9.4	0.17	56	9.4	0.17	56	9.4	0.17
Sem ±	1.085	0.88	0.02	1.09	0.88	0.019	1.085	0.88	0.02
Grand mean	3.36	16.26	19.3	3.36	16.26	19.33	3.36	16.26	19.3
FIM∗(IWAL∗NFRA) (P)	0.01	0.0001	0.01	0.01	0.0001	0.001	0.01	0.0001	0.01

**Note:** Mean results in the same column followed by different letters are significantly different in the least significant difference (LSD) tests at *P* < 0.05 level, NFAR is Nitrogen fertilizer application rate, and MAD is management allowable depletion.

The interaction results of the furrow irrigation method and management allowable depletion (MAD) with N-fertilizer application rate (NFAR) on mean marketable fruit yield ranged from 2.3 tons ha^−1 to 43.9 tons ha−1. The maximum marketable yield from interaction factors (43.9 tons ha−1) was recorded from CFIM, 100 % MAD with 100 % NFAR control treatment. The first four highest mean marketable fruit yields, followed by control treatments were 33.5 tons ha−1, 32.9 tons ha−1, 31.6 tons ha−1, and 29.7 tons ha−1. These four highest mean marketable fruit yields were recorded from the treatments of 100 % MAD, 150 % NFAR with CFIM; 100 % MAD, 100 % NFAR with AFIM; 75 % MAD, 100 % NFAR with CFIM; and 100 % MAD, 100 % NFAR with FFIM, respectively. However, the first most minimum marketable fruit yield recorded values of 2.3 tons ha−1, 3.2 tons ha−1, and 4.3 tons ha−1^ were recorded from the treatment 50%MAD, 0%NFAR under FFIM, AFIM, and CFIM respectively (Table 3).

The mean unmarketable yield of tomatoes ranges from 0.8 to 8 tons ha^−1^. The maximum unmarketable tomato yield of 8 tons ha^−1^ was recorded at 50 % MAD with 0 % NFAR and Fixed furrow irrigation method (FFIM). The first three maximum mean unmarketable fruit yields followed by 50 % MAD with 0 % NFAR treatment were 5.36 tons ha^−1^, 5 tons ha^−1^, and 4.48 tons ha^−1^. These results were recorded from the treatments of 50 % MAD with 50 % NFAR, 50 % MAD with 150 % NFAR, and 75 % MAD with 0 % NFAR, respectively (Table 3).

The mean total tomato results from the interaction of furrow irrigation methods and irrigation water application level with N-fertilizer application rate factors range from 6.3 to 45.2 tons ha^−1^. The maximum total fruit yield of 45.2 tons ha^−1^ was recorded from the control treatment (100 % MAD with 100 % NFAR under CFIM). Next, to control treatment results, the first most preferable highest total fruit yields were 34.8 tons ha^−1^ from the treatment 100 % MAD with 150 % NFAR under CFIM. The second most total tomato yield was 33.9 tons ha^−1^ from both the treatments of 100 % MAD with 100 % NFAR under AFIM and 75 % MAD and 100 % NFAR under CFIM. Furthermore, the third total yield result was 30.5 tons ha^−1^ from treatments of 100 % MAD with 100 % NFAR under FFIM (Table 3). In contrast, the 50 % MAD with 0 % NFAR under fixed furrow irrigation methods notably produced a low yield of total fruit yield of 6.3 tons ha^−1^.

### 3.3 Economic feasibility analysis for tomato crop production

The economic analysis revealed distinct profitability trends among the treatment combinations. The treatments that generated the highest profits were CFIM with 100 % MAD and 100 % NFAR, and AFIM with 100 % MAD and 100 % NFAR, both yielding a net profit of $32. Additionally, CFIM with 100 % MAD and 50 % NFAR followed closely, with a profit of $28. These results suggest that higher nitrogen fertilizer application rates, when combined with a complete water supply (100 % MAD), significantly enhance tomato yields and, consequently, economic returns. Conversely, the lowest profit treatments were identified as FFIM with 50 % MAD and 50 % NFAR, resulting in a loss of $11, and FFIM with 50 % MAD and 0 % NFAR, leading to a loss of $8. AFIM with 50 % MAD and 0 % NFAR also showed negative returns with a loss of $4 as shown in Table 4. The poor performance of these treatments highlights the detrimental impact of inadequate irrigation and nitrogen supply on profitability.

**Table 4:** Economic perform results for each treatment.

Treatments	Conventional furrow irrigation method (CFIM)			Alternate furrow irrigation method (AFIM)			Fixed furrow irrigation method (FFIM)		
	Yield (Output Cost)	Total Input Cost ($)	Profit (Net Return)($)	Yield (Output Cost)	Total Input Cost ($)	Profit (Net Return)($)	Yield (Output Cost)	Total Input Cost ($)	Profit (Net Return)($)
100%MAD ∗ 0%NFAR	35	11	24	31	11	20	29	11	18
100 % MAD ∗ 50%NFAR	30	11	19	25	11	14	25	11	14
100%MAD ∗ 100%NFAR	11	11	0	7	11	−4	3	11	−8
100%MAD ∗ 150%NFAR	40	12	28	36	12	24	31	12	19
75%MAD ∗ 0%NFAR	35	12	23	31	12	19	22	12	10
75%MAD ∗ 50%NFAR	15	12	3	15	12	3	1	12	−11
75%MAD ∗ 100%NFAR	45	13	32	45	13	32	34	13	21
75%MAD ∗ 150%NFAR	40	13	27	34	13	21	21	13	8
50%MAD ∗ 0%NFAR	31	13	18	25	13	12	15	13	2
50%MAD ∗ 50%NFAR	42	13.5	28.5	39	13.5	25.5	32	13.5	18.5
50%MAD ∗ 100%NFAR	39	13.5	25.5	34	13.5	20.5	20	13.5	6.5
50%MAD ∗ 150%NFAR	30	13.5	16.5	27	13.5	13.5	10	13.5	−3.5

**Note:** NFAR is Nitrogen fertilizer application rate, and MAD is management allowable depletion.

## 4 Discussion

This experimental study investigated the integrated effects of CFIM, AFIM, FFIM, management allowable depletion (MAD), and nitrogen fertilizer application rates (NFAR) are crucial for maximizing tomato yield in semi-arid regions of southern Ethiopia. Their combined impact on various growth parameters and yield components of tomato crops over two consecutive years. Our findings highlight the crucial interplay between these factors in optimizing tomato production under water-limited conditions. Notably, the findings underscore the significant influence of these factors on plant height, number of branches, root depth, and yield components such as marketable and unmarketable fruit yields.

Firstly, regarding plant height, the integrated irrigation method, MAD level, and nitrogen rate treatments significantly influenced tomato crop height. The analysis revealed a clear pattern wherein conventional furrow irrigation techniques and higher levels of MAD application combined with optimal nitrogen fertilizer application rates as control treatment led to the tallest tomato plants. Our study found that the tallest plants followed by control treatment were observed under moderate irrigation with alternative furrow irrigation and optimal nitrogen application. Conversely, the shortest plants were observed under fixed furrow irrigation methods and lower irrigation water levels with surplus and without nitrogen fertilization. These results suggest that adequate irrigation and nitrogen fertilization positively impact plant height, likely through enhanced soil moisture availability and improved nutrient uptake, facilitating vegetative growth. This finding supports the work of [33] who demonstrated that optimal irrigation and fertilization practices result in increased vegetative growth in tomatoes.

The number of branches per tomato plant is an important agronomic trait that affects light interception and photosynthetic efficiency. Our results showed that the highest branch number followed by the control treatment was achieved under 75 % MAD of moderate irrigation and optimal nitrogen rate (100 % NFAR) under the conventional furrow irrigation method. Moreover, 100 %MAD, alternative furrow irrigation method, and 100 % NFAR are the best preferable integration applications without a reduction of the branches number as compared to the control. However, the deficit or surplus nitrogen fertilizer rate application with and without full application of MAD and conventional or other furrow irrigation methods hurts the number of branches. Similar, findings were reported by Ref. [34] who noted that a balanced water and nutrient supply enhances branching by improving the overall canopy structure and light-use efficiency of tomato plants.

Root depth is a critical factor for water and nutrient uptake, especially in semi-arid regions. The integrated furrow irrigation methods, irrigation water management allowable depletion, and nitrogen application rate treatments significantly affected tomato root depth. The deepest roots were observed under conditions of moderate irrigation and optimal nitrogen levels. Deep rooting allows plants to access water and nutrients from deeper soil layers, enhancing their resilience to drought stress. Our results showed that the highest root depth followed by the control treatment was achieved under 100 % MAD and 150 % NFAR under the conventional furrow irrigation method. However, this treatment NFAR was half percent surplus which shows that it needs a high quantity of N fertilizer and cost. Instead of this treatment, we used 75 % MAD of moderate irrigation, and the optimal nitrogen rate (100 % NFAR) under the conventional furrow irrigation method was preferred. Moreover, 100 %MAD, alternative furrow irrigation method, and 100 % NFAR are the best preferable integration applications without a reduction of the branches number as compared to the control. However, the deficit nitrogen fertilizer rate application with and without full application of MAD and conventional or other furrow irrigation methods hurts the root depth of the tomato. This finding corroborates the work of [35,36], who found that optimal irrigation and fertilization promote deeper root growth in tomatoes, improving their ability to withstand water deficits.

The integrated effects of MAD level or irrigation water level, furrow irrigation methods, and nitrogen fertilizer rate significantly influenced marketable tomato yield. Our findings demonstrate that optimal irrigation combined with efficient furrow irrigation methods and appropriate nitrogen levels can substantially enhance marketable yields. Specifically, next to control treatment 100 % MAD irrigation levels paired with alternative furrow irrigation and an optimal nitrogen rate are preferably applicable without marketable yield reduction. Secondly, AFI with 100 % MAD and 100 kg/ha N fertilizer applied and/or 75%MAD moderate irrigation levels paired with conventional furrow irrigation and an optimal nitrogen rate can significantly improve tomato yield and quality. These can be a 25 % water-saving strategy without significant yield loss in semiarid tomato farms. Similarly, several scholars have reported the effects of irrigation and nutrients on marketable tomato yield and saving each other [37]. Our results also confirmed that the marketable tomato yield increases the balance of irrigation water using different irrigation levels with 100 % nitrogen fertilizer [38,39] However, the interaction of the deficit or surplus nitrogen fertilizer rate application with and without full application of MAD and conventional or other furrow irrigation methods hurts the marketable yield. This result is in line with the research by Ref. [34], who found that precise irrigation and nitrogen management improved water and nutrient uptake efficiency, leading to higher marketable tomato yields. Moreover, Studies have shown that precise water management enhances fruit quality and quantity by preventing stress conditions that can lead to poor fruit set and size reduction [[10], [11], [12],^36^].

Unmarketable tomato yield was also negatively affected by the integration of irrigation methods, MAD levels, and NFAR management practices. Excessive or insufficient irrigation water and N fertilizer can lead to conditions such as blossom-end rot and cracking, which reduce marketable yield. Our results indicated that unmarketable yield was lowest under conditions of optimal water and nitrogen application. Several scholars reported the combined effects of irrigation and nitrogen management on tomato production in Ethiopia and throughout the world [37,40,41] Their results indicated that precise control of irrigation water and nutrients applied could achieve higher yields and reduce environmental impacts, particularly in water-scarce environments. However, the integration of deficit or surplus nitrogen fertilizer rate application with and without full application or deficit of MAD and conventional or other furrow irrigation methods increases the unmarketable yield of tomatoes. This is consistent with the findings of [34] who observed that irrigation water and nutrient supply minimize physiological disorders and pest infestations, thus reducing the proportion of unmarketable fruits.

The total tomato yield, comprising both marketable and unmarketable components, was highest under the control combined treatment (100 % MAD with 100 % NFAR under CFIM). This comprehensive approach ensures that plants receive adequate water and nutrients throughout their growth cycle, leading to increased overall productivity. The integration of 75%MAD with 100%NFAR under conventional, and 100%MAD with 100 NFAR under alternative furrow irrigation was the most preferable without loss of Nitrogen fertilizer loss and 25 % irrigation water saved without tomato yield reduction. The research conducted by Refs. [42,43]demonstrated that alternate furrow irrigation combined with reduced nitrogen application significantly improved water use efficiency and tomato yields compared to conventional furrow irrigation. Their findings align with this study's results, which also highlight the superior performance of alternate furrow irrigation in conserving water while maintaining high yields. However, the interaction of the deficit nitrogen fertilizer rate application with and without full application of MAD and conventional or other furrow irrigation methods causes the total yield to decrease. The effectiveness of alternative furrow irrigation combined with optimized nitrogen application in enhancing tomato yield and quality [13]. Additionally, the critical role of balanced water and nutrient supply in reducing unmarketable yield and improving overall crop performance [44,45].

Nitrogen leaching is a critical environmental concern, particularly in irrigated agricultural systems where excessive nitrogen application combined with improper water management can result in nitrogen being washed below the root zone. This not only reduces the nitrogen available to plants but can also contaminate groundwater sources, contributing to water pollution. The application of nitrogen in excess, particularly in the form of urea, increases the risk of nitrate leaching into the water table, which has been associated with health risks such as methemoglobinemia or "blue baby syndrome" [31]. Implementing precise nitrogen application strategies, such as split application and adjusting rates based on crop demand and soil moisture, helps mitigate these risks. Moreover, the use of alternate or fixed furrow irrigation methods, which reduce the amount of water applied, could further reduce the risk of leaching by minimizing excessive water percolation through the soil profile by controlling soil moisture levels.

The results underscore the critical role of both irrigation management and nutrient supply in the economic viability of tomato production. Specifically, treatments such as CFIM with 100 % MAD and 100 % nitrogen fertilizer application rate (NFAR) demonstrated a profit of $32. while AFIM exhibited similar economic performance, reinforcing the notion that integrated water and nutrient management practices are crucial for maximizing agricultural output [38,46]. Conversely, the Fixed Furrow Irrigation Method (FFIM) at lower moisture and nitrogen levels showed negative profitability($2), highlighting the inefficiency of this approach under suboptimal conditions, which aligns with findings by Refs. [46,47]. These results underscore the importance of adopting advanced irrigation and fertilization strategies to enhance crop yields and ensure financial sustainability in tomato production systems [22].

The influence of environmental factors was not directly considered as variable factors in this study. While the primary focus was on the selected irrigation and fertilization treatments, the surrounding environmental conditions such as temperature fluctuations, rainfall variability, and soil characteristics can significantly impact the outcomes of these interventions. For instance, elevated temperatures may exacerbate evapotranspiration rates, leading to increased water demand and possibly affecting yield if not adequately managed [48]. Furthermore, soil properties, including texture and organic matter content, influence water retention and nutrient availability, thereby modulating plant responses to the applied treatments [10]. Additionally, the interactions with other management practices like pest control, crop rotation, and soil amendments are relevant for optimizing tomato yield but were not evaluated as factors in this study. These practices can enhance the efficiency of nitrogen uptake and mitigate pest pressures that could adversely affect yield [37]. Ignoring these potential interactions may limit the comprehensive understanding of how the tested irrigation and fertilization strategies perform in real-world scenarios. Moreover, the extrapolation of findings to larger agricultural systems is another consideration. Results derived from localized studies and applicable to the same climate condition, and soil properties. However, it may not be universally applicable due to inherent variations in climate, soil types, and farming practices across different regions [8,49].

## 5 Conclusion

The combined impact of the considered factors on various growth parameters and yield components of tomato crops over two consecutive years highlights the crucial interplay necessary for optimizing tomato production under water-limited conditions. Notably, the findings underscore the significant influence of these factors on plant height, number of branches, root depth, and yield components such as marketable and unmarketable fruit yields. Our results showed that moderate irrigation combined with alternative furrow irrigation methods and optimal nitrogen application resulted in the tallest plants, highest branch numbers, and deepest root systems. In terms of yield components, optimal irrigation, and nitrogen application were critical in maximizing marketable tomato yield while minimizing unmarketable yield. The integration of 75%MAD with 100%NFAR under conventional, and 100%MAD with 100 NFAR under alternative furrow irrigation proved to be a water-saving and without loss of Nitrogen fertilizer loss strategy without significant yield loss. By adopting these practices, farmers will achieve higher productivity and sustainability in tomato cultivation, addressing the challenges posed by water scarcity and nutrient limitations while increasing their economic performance. The findings provide a solid foundation for developing best management practices that can be tailored to specific local conditions, ultimately contributing to food security and economic development in semi-arid regions. The farmers adopt integrated water and nutrient management strategies, focusing on effective irrigation and fertilization practices, to optimize tomato crop yields and enhance economic viability. Additionally, future studies should account for environmental factors, integrate other management practices, and incorporate precision agriculture technologies to enhance water, nutrient use efficiency, and tomato yield in semi-arid regions like Southern Ethiopia.

## CRediT authorship contribution statement

**Edmealem Temesgen Ebstu:** Writing – original draft, Visualization, Validation, Supervision, Software, Resources, Project administration, Methodology. **Mekuanent Muluneh:** Writing – review & editing, Investigation, Funding acquisition, Formal analysis, Data curation, Conceptualization.

## Data availability statement

Data will be made available on request. For requesting data, please write to the corresponding author.

## Declaration of competing interest

The authors declare that they have no known competing financial interests or personal relationships that could have appeared to influence the work reported in this paper.
